# The mediating effect of social support on the relationship between intimacy and perceived partner responsiveness in endometriosis women of childbearing age

**DOI:** 10.3389/fpsyg.2024.1437335

**Published:** 2025-01-06

**Authors:** Jia Chen, Yuanjing Ge, Xiaohong Jin, Haiyan Huang, Xiao Shan, Xujuan Xu

**Affiliations:** ^1^Department of Gynecology, Affiliated Hospital of Nantong University, Nantong, China; ^2^School of Nursing and Rehabilitation, Nantong University, Nantong, China; ^3^Department of Human Resource, Affiliated Hospital of Nantong University, Nantong, China; ^4^Department of Nursing, Affiliated Hospital of Nantong University, Nantong, China

**Keywords:** social support, intimacy, perceived partner responsiveness, women with endometriosis, childbearing age

## Abstract

**Purposes:**

The mediating effect of social support on the relationship between intimacy and perceived partner responsiveness (PPR) was examined among the endometriosis women of childbearing age.

**Method:**

201 endometriosis (EM) women of childbearing age were investigated in the gynecology outpatient clinic and ward of a tertiary general hospital in Nantong City, Jiangsu Province. Intimacy, PPR, and social support were measured by using the scales of Quality of Relationship Index (QRI), PPR Scale (PPRS), and Social Support Rating Scale (SSRS). A four-step hierarchical regression analysis was conducted.

**Results:**

Positive correlations were found among intimacy and subjective support, support utilization, and PPR. Mediation analysis indicated a partial mediating effect of support utilization on the relationship between intimacy and PPR.

**Conclusion:**

There is a positive correlation between intimacy and PPR in EM patients of childbearing age, and the support utilization plays a partial mediating role.

## 1 Introduction

Endometriosis (EM) is a common disease among women of childbearing age, which refers to the presence of endometrial glands and stroma with growth function in other parts of the body other than the uterine cavity. Ectopic endometrium is most commonly found in adjacent tissues and organs such as ovaries, uterosacral ligament, uterine myometrium, pelvic floor peritoneum, and rarely in extrapelvic organs such as gastrointestinal tract, urinary tract, lungs, nasal mucosa, and brain (Andres et al., 2020; Davis and Goldberg, 2017; Machairiotis et al., 2013). The main symptoms are lower abdominal pain and dysmenorrhea, menstrual abnormalities, infertility, discomfort during sexual intercourse, and other specific symptoms, 40–60% of patients have concurrent severe pain or chronic pelvic pain, and 20–30% of patients have reduced fertility (Leng and Shi, 2018). Studies have shown that EM symptoms adversely affect the relationship between women and their partners (Bień et al., 2020). Some male partners believe that a number of factors, such as women’s painful intercourse, inability to have children, disease symptoms, side effects from treatment, shifting family roles, and reduced finances, have strained their intimacy (Culley et al., 2017; Hudson et al., 2020). Many researchers have focused on the physical and psychological problems that exist in EM women of childbearing age, but few researchers have studied the mediating effect of social support on the relationship between intimacy and perceived partner responsiveness (PPR).

PPR is the degree to which individuals perceive their partner’s caring and understanding of their thoughts and feelings (Reis, 2012; Reis et al., 2004). The basic function of PPR is to reduce negative emotions and increase feelings of security (Slatcher and Schoebi, 2017; Slatcher and Selcuk, 2017), thereby fulfilling the basic need for belonging and connectedness (Baumeister and Leary, 2017). Tosyali and Harma (2024) noted a more pronounced positive association between PPR and subjective health. Hillmer et al. (2021) confirmed that PPR can moderate pain. Soares et al. (2021) concluded that PPR has a greater effect in stress relief and promotion of mental health during the COVID-19 pandemic. Fenech et al. (2022) found that higher PPR is positively associated with improved subjective sleep during a study of couples coping with early stage breast cancer. Xie et al. (2023) found that when husbands conduct higher negative partner interactions and lower positive partner interactions, wives develop negative PPR and have lower sleep quality in the night. Rice et al. (2020) suggested that individuals with high PPR also exhibit lower partner-specific attachment anxiety and partner-specific avoidance. Most studies suggest that higher intimacy may enhance understanding, communication, and support (Van Niekerk et al., 2021), and the intimacy can be improved when women perceive their partners as supportive, empathetic, and caring.

As an effective resource for improving the lifestyles of the supported people (Chen et al., 2007), social support predicts all-cause mortality, and when women, as supported, receive higher levels of social support from their partners, their physical and mental health can be improved, which can lead to lower mortality rates (Kroenke et al., 2012). Physical symptoms such as pain, infertility, and fatigue are the main factors affecting the depression and anxiety in EM, causing a great deal of mental and physical stress, which may lead to stigma. EM patients who have a higher need for social support also experience poorer quality of life and self-esteem (Matías-González et al., 2022), which can further intensify the avoidance of social activities, and their perceptions of social support can also be significantly affected. As some respondents do not have a clear diagnosis, they feel that some people do not believe they are unwell, which increases their sense of isolation (Rea et al., 2020). Only 20% of the population are aware of EM because of the diversity of symptoms and the fact that many people believe that “menstruation is normal.” Therefore, many patients do not receive the social support they want and are in need of a positive social environment where more people are aware of the symptoms of EM and share information about them. Good social relationships are important for health (Schick et al., 2022), especially when social support comes from a partner, and social support is positively associated with personal well-being (Boyers and Simpson Rowe, 2018).

Intimacy has a positive impact on recovery from various physical and mental illnesses (Robles et al., 2014). EM has a negative impact on intimacy, and the support of a partner is very important for women with EM (Facchin et al., 2021). It has also been argued that this process of facing the disease together can lead to an improved intimacy between couples (Culley et al., 2017). In 1977, Raschke (1978) proposed that social support refers to people’s perceived concern and support from others, social support has a beneficial buffering effect on the stress experienced by individuals in the stressful situations, and a key factor is the individuals’ perception that others will provide them with appropriate supports, which will alleviate the individuals’ emotional and physiological responses to stress. Thus, we can hypothesize that the social support is positively correlated with PPR.

**Figure 1:**
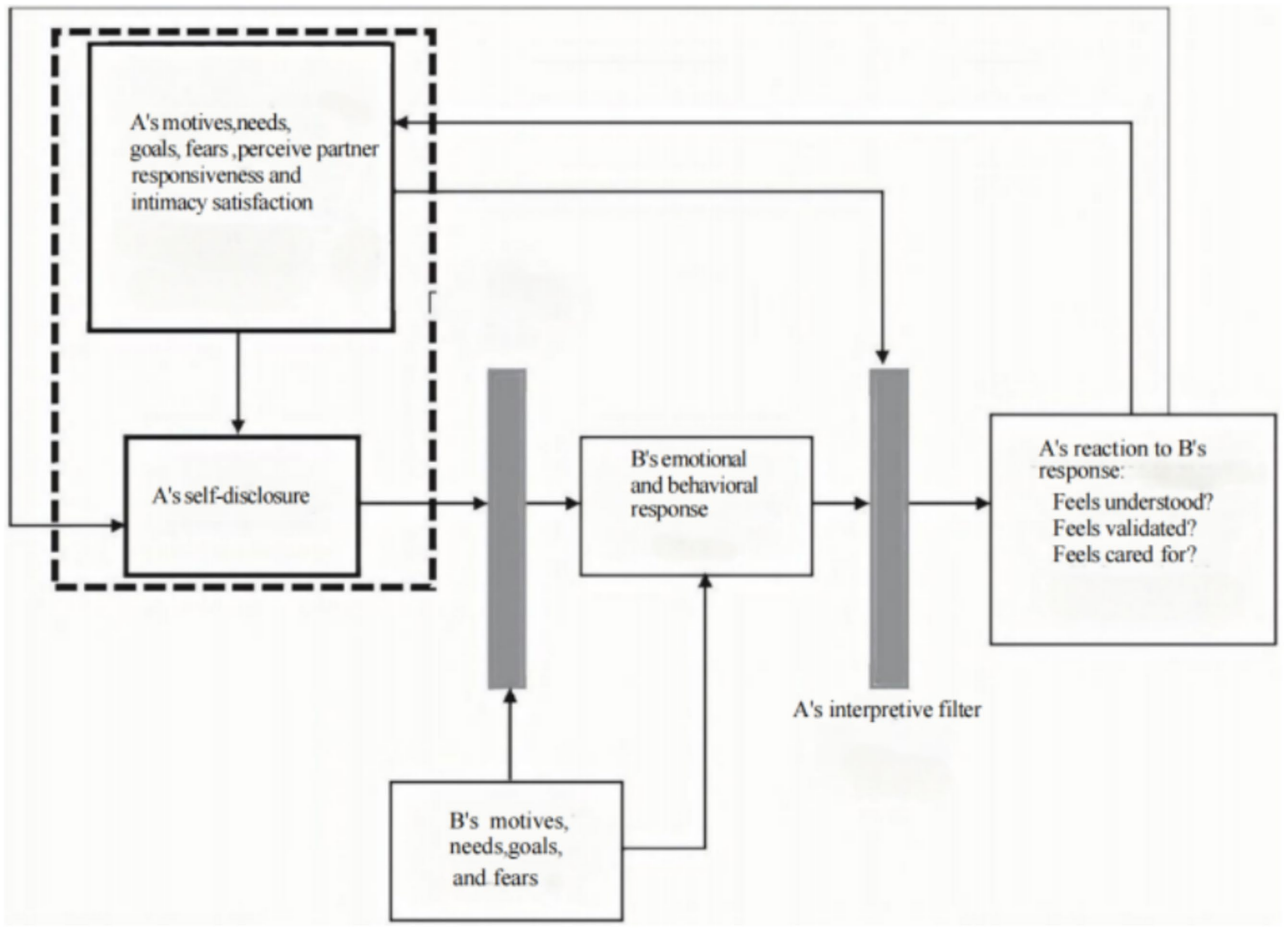
IPM model of intimacy.

PPR is one of the basic elements of the interpersonal process model (IPM) of intimacy (see Figure 1), which was proposed by Reis and Shaver (1988), who argue that the establishment of intimacy is a dynamic developmental process in which self-representation and spousal responsiveness are key components (Laurenceau et al., 1998). The model reveals that the intimacy development process consists of the following 3 steps. (1) One spouse (the speaker A) exchanges information with the other spouse (the listener B); (2) B responds to what A has communicated and conveys his or her understanding and support to A; and (3) A perceives the B’s response. A’s intimacy continues to increase, B must respond to A in a variety of ways; he/she must understand the communication content of A, accept and recognize the value of A, and report positive attitudes toward A in order for A to perceive his/her communication with B as responsiveness. As this pattern of communication is repeated, the intimacy between the two spouses is enhanced.

Emotional support is one of the core elements in intimacy. When individuals feel the care, understanding and support from their partners in intimacy, their emotional needs are met, and the intimacy satisfaction is increased. At the same time, information sharing between partners helps individuals better cope with challenges and difficulties in life, and provides practical help and mutual support when needed, all of which can enhance mutual trust and dependence, and make individuals more likely to feel understood and cared for, thus enhancing the positive PPR. Of course, PPR can enhance individuals’ intimacy satisfaction. This is because when individuals have perceived positive evaluations and expectations from their partners, they are more willing to invest time and energy in maintaining and growing this relationship, and are more willing to share their needs and feelings with their partners. In this theoretical framework, the social support connects intimacy and PPR. By providing emotional, informational, and practical support, social support can enhance the individual’s sense of trust and dependence, and promote PPR, which in turn can promote the healthy development of intimacy.

## 2 Research hypothesis and aim

In this study, which focused on the effects of intimacy on EM women of child bearing age, we formulated the following hypotheses:

(1) Intimacy with partner and PPR are positively correlated in EM women of childbearing age.
(2) Social support exert a mediating effect on the relationship between intimacy with partner and PPR in EM women of childbearing age.

**Figure 2:**
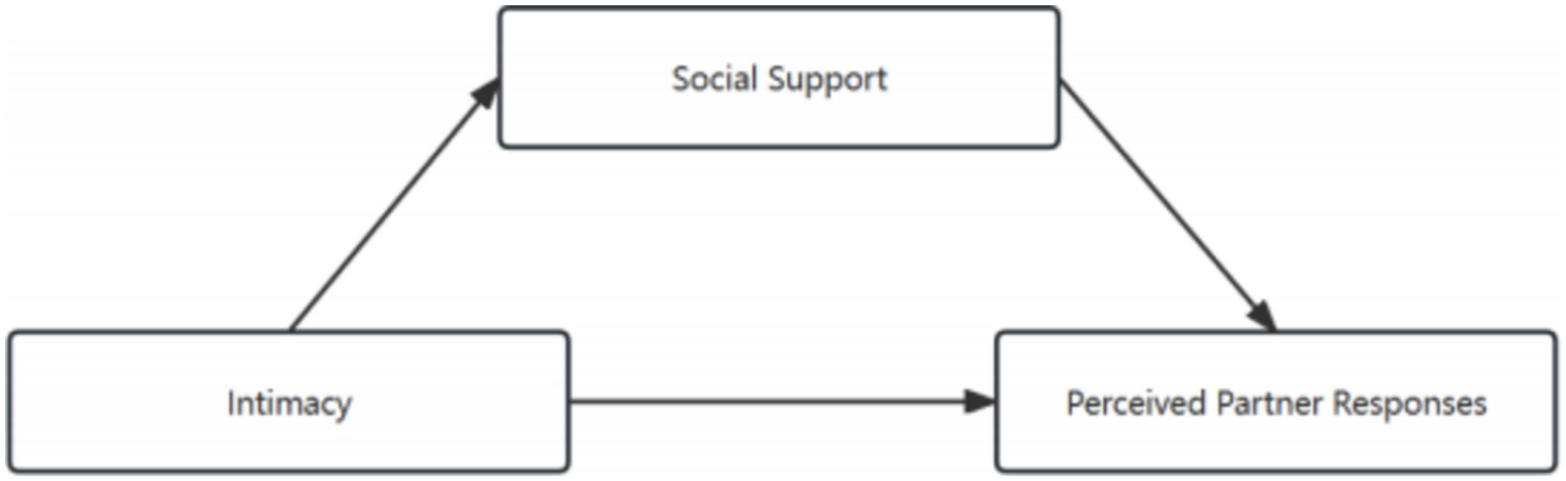
Theoretical mediation model.

This study aims to explore not only the mediation effect of social support on the relationship between intimacy and PPR but also a direct correlation between these two variables. Which are major issues of long-term and far-reaching significance to enable EM women of childbearing age to cope with the onset of the disease in a positive manner (see Figure 2).

## 3 Methodology

### 3.1 Participants

This study was conducted using a convenience sampling method from November 2022 to August 2023 in the gynecology outpatient clinic and ward of a tertiary general hospital in Nantong City, Jiangsu Province, China. This study was reviewed by the Ethics Committee of Affiliated Hospital of Nantong University (2023-K171-01). Inclusion criteria: (1) female patients who aged 18–49 years old and had normal menstruation; (2) those who met the diagnostic criteria of EM; (3) those who had in a serious and stable heterosexual intimate relationship; (4) those with normal understanding and recognizing abilities and an ability to communicate clearly; (5) those who had signed an informed consent form, with a willingness to participate in the survey. Exclusion criteria: (1) patients who suffered from mental illness and were unable to complete the questionnaire independently; (2) those with other complications.

### 3.2 Instruments

#### 3.2.1 PPR scale (PPRS)

The PPRS is used to assess the degree of PPR in subjects, and was developed by Reis (2012). The Chinese version was revised by Yang et al. (2019). This scale has a single structural dimension with 12 items, and a 7-point Likert rating method was adopted, with 1 to 7 representing “not at all consistent” to “completely consistent.” The total score ranges from 12 to 84, and a higher score indicates a higher PPR. The scale is designed for adults over the age of 18 who have been in a relationship for more than 6 months. This scale has a Cronbach’s alpha coefficient of 0.90 and a reliability of 0.73, which has good reliability and validity.

#### 3.2.2 Social support rating scale (SSRS)

This study used the Chinese version of the Social Support Rating Scale developed by Xiao (1994) to measure social support. The scale consists of 10 items in 3 dimensions: subjective support (4 items), objective support (3 items) and support utilization (3 items). The total score ranges from 12 to 66, which is the sum of the scores of each item, and a higher score means that the respondent receives more social support; where a total score of ≤22 is considered a low level, a total score of 23–44 is considered a medium level, and a total score of ≥45 is considered a high level. The Cronbach’s alpha coefficient for this scale was 0.896.

#### 3.2.3 Quality of relationship index (QRI)

The QRI is a commonly used tool for measuring intimacy satisfaction. This scale was developed by Patrick et al. (2007) and its Chinese version was translated and revised by Qiu (2010). It consists of 6 items, which effectively cover all aspects involved in intimacy, including emotional experience, intimacy, commitment, and permanence, etc. The Cronbach’s alpha coefficient for this scale was 0.91.

### 3.3 Data analysis

All analyses were performed using SPSS 23.0 (IBM) and MPLUS 8.3. Initially, descriptive analyses were conducted on demographic characteristics, social support, intimacy, and PPR. The data with a normal distribution were described as means ± standard deviations, and the qualitative data were described as frequencies and percentages. Subsequently, correlational analyses were conducted to estimate the relationship among social support, intimacy, and PPR. Thirdly, regression analyses were conducted to assess the role of social support as a mediator (M) between independence (intimacy) and dependence (PPR). The bootstrap was set to 5,000 times, and 95% confidence intervals were reported using bootstrapping The significance level for tests was set at *α* = 0.05.

## 4 Results

### 4.1 General situation of research object

A total of 224 questionnaires were distributed, 201 valid questionnaires were recovered, with an effective recovery rate of 89.73%0.201 EM women of childbearing age who met the inclusion criteria were selected, and all subjects were informed of the purpose of this study and volunteered to take part in this study. All of the subjects included were Han Chinese. 50% of them were in the age group of 31–40 years old; 74 were undergraduates, accounting for 36.8%; most of them had full-time job; 11.9% of them self-payed for medical expenses; 64 had an income of 2000–4,000 yuan, accounting for 31.8%; and the majority of them were cohabiting with their partners. During the course of the disease, 134 complained of pain, 69 had symptoms of masses, and 17 had symptoms of infertility; the vast majority chose surgical treatment; 60 had requests for fertility, of whom 35 had not yet given birth, and 8 chose to use assisted reproductive technology, see details in Table 1.

**Table 1:** Baseline information of the study subjects.

Variables	Frequency
Age group (years)	
18–25	10 (5)
26–30	22 (10.9)
31–40	100 (49.8)
41–49	69 (34.3)
Nationality	
Han	201 (100)
Religious faith	
Non	187 (93)
Buddhism	10 (5)
Others	4 (2)
Educational level	
Primary school and below	5 (2.5)
Junior high school	50 (24.9)
Senior high school or technical secondary school	28 (13.9)
Junior college	41 (20.4)
Undergraduate	74 (36.8)
Postgraduate and above	3 (1.5)
Current employment status	
Unemployed	22 (10.9)
Part-time job	9 (4.5)
Full-time job	125 (62.2)
Sick leave	12 (6)
Others	33 (16.4)
Place of residence	
Villages and towns	85 (42.3)
County town	62 (30.9)
Prefecture-level city/provincial capital city	54 (26.9)
Average monthly income (yuan/month)	
<2000	31 (15.4)
2000–4,000	64 (31.8)
4,001–6,000	46 (22.9)
>6,000	60 (29.9)
Payment method of medical expenses	
Self-paying	
No	177 (88.1)
Yes	24 (11.9)
Basic medical insurance for urban workers	
No	56 (27.9)
Yes	145 (72.1)
Rural cooperative medical insurance	
No	171 (85.1)
Yes	30 (14.9)
Medical insurance for urban residents	
No	187 (93)
Yes	14 (7)
Commercial insurance	
No	189 (94)
Yes	12 (6)
Others	
No	197 (98)
Yes	4 (2)
Living situation with partner	
Live together	150 (74.6)
Live in different places	51 (25.4)
Pain	
No	67 (33.3)
Yes	134 (66.7)
Mass	
No	132 (65.7)
Yes	69 (34.3)
Infertility	
No	184 (91.5)
Yes	17 (8.5)
Pain score (point)	
0	46 (22.9)
1	7 (3.5)
2	18 (9)
3	24 (11.9)
4	18 (9)
5	17 (8.5)
6	17 (8.5)
7	16 (8)
8	18 (9)
9	8 (4)
10	12 (6)
Duration of reaching a definite diagnosis	
Less than 6 moths	41 (20.4)
6 moths-1 year	36 (17.9)
1 year-3 years	58 (28.9)
3 years-5 years	15 (7.5)
More than 5 years	51 (25.4)
Treatment method (Regular follow-up)	
No	116 (57.7)
Yes	85 (42.3)
Treatment method (drug treatment)	
No	149 (74.1)
Yes	52 (25.9)
Treatment method (surgical treatment)	
No	60 (29.9)
Yes	141 (70.2)
Treatment method (assisted reproductive technology)	
No	193 (96)
Yes	8 (4)
Reproductive needs	
Present	60 (29.9)
Absent	141 (70.2)
Number of pregnancies (times)	
0	28 (13.9)
1	86 (42.8)
2	52 (25.9)
3	26 (12.9)
4	8 (4)
6	1 (0.5)
Number of births (times)	
0	36 (17.9)
1	142 (70.6)
2	20 (10)
3	2 (1)
5	1 (0.5)
Number of surviving children	
0	35 (17.4)
1	146 (72.6)
2	19 (9.5)
3	1 (0.5)
Past medical history	
Non	177 (88.1)
Diabetes	3 (1.5)
Hypertension	7 (3.5)
Reproductive system disease	3 (1.5)
Others	11 (5.5)
Family medical history	
None	159 (79.1)
Hypertension	29 (14.4)
Diabetes	8 (4)
Others	5 (2.5)

### 4.2 Measurement scores and correlation analysis

The subjects included in this study had an intimacy satisfaction score of 33.3 ± 7.6, an objective support score of 6.92 ± 3.37, a subjective support score of 25.27 ± 4.54, a support utilization score of 7.91 ± 2.15, and a PPR score of 57.37 ± 12.34. As shown in Table 2, the intimacy was significantly correlated with all subjective support, support utilization, and PPR (*p* < 0.001), of which, the correlation coefficient between the intimacy and the subjective support was highest (0.446); while PPR was not only correlated with intimacy, but also significantly correlated with the subjective support and support utilization (*p* < 0.001), and the correlation coefficient between PPR and subjective support was higher (0.277).

**Table 2:** Score and correlation between variables.

	M ± SD	1	2	3	4	5
Intimacy	33.3 ± 7.6	1				
Objective support	6.92 ± 3.37	0.028	1			
Subjective support	25.27 ± 4.54	0.446***	0.093	1		
Support utilization	7.91 ± 2.15	0.283***	0.194**	0.386***	1	
Partner responsiveness	57.37 ± 12.34	0.47***	0.052	0.277***	0.262***	1

*p < 0.05; **p < 0.01; ***p < 0.001.

### 4.3 Analysis of mediating effect of social support on intimacy and PPR

The mediating role of social support between intimacy and PPR was examined through mediation analyses. The results are presented in Table 3. The total effect of intimacy on PPR was significant, with B (unstandardized coefficient) of 0.76, standard error (SE) of 0.10, standardized beta coefficient (*β*) of 0.47, and *p* < 0.001. The direct effect was reduced when support utilization was included as a mediator, with B of 0.45, SE of 0.09, *β* of 0.28, and *p* < 0.01. Support utilization played a mediating role between intimacy and PPR, with a mediation effect ratio of 0.085 (95% CI: 0.006–0.2).

**Table 3:** Mediation results.

Mediator	Mediation analysis paths		Estimated	95% CI		*p*
				LLCI	ULCI	
Objective support	ACME		0.002	−0.013	0.020	0.780
	ADE		0.761	0.559	0.980	<0.001
	Prop.	Mediated	0.002	−0.016	0.030	0.780
Subjective support		ACME	0.061	−0.035	ACME	0.061
	ADE		0.701	0.441	0.950	<0.001
	Prop.	Mediated	0.080	−0.043	0.260	0.230
Support utilization		ACME	0.065	0.005	0.140	0.034
	ADE		0.698	0.459	0.920	<0.001
	Prop.	Mediated	0.085	0.006	0.200	0.034

ADE, average direct effect; ACME, average causal mediated effect.

## 5 Discussion

This study focused on EM women of childbearing age, all of them were in a stable intimate relationship, with varying degrees of pain and infertility. Most of the patients worked full-time but generally had low incomes. Previous studies on the mediation of social support have focused more on populations with chronic diseases (Yeung et al., 2022), but less on women with gynecological diseases, especially those of childbearing age.

The PPR score for EM women of childbearing age in this study was 57.37 ± 12.34, which was higher relative to other studies. In other similar studies using the PPRS scale, we found that the PPR score of cervical cancer patients of childbearing age was 54.04 ± 8.01 (Yuan et al., 2023) and that of breast cancer patients of childbearing age was 40.11 + 5.15 (Sun et al., 2022). The reason for this is that although EM is prone to recurrence, has a biologic behavior similar to tumor invasion and metastasis, also often involves other organs, and shares pathophysiologic characteristics with cancer, it is not a malignant tumor and is not life-threatening (Wilbur et al., 2017). Compared to malignant tumors such as cervical and breast cancers, EM women and their partners may have lower stress and are more able to cope with the disease in a positive way, so the partners usually show a high level of PPR. The present study found a positive correlation between intimacy and PPR in EM women with of childbearing age, which is consistent with the findings of Champagne and Muise (2022) and Mohammadi et al. (2018). Attaky et al. (2020) concluded that PPR fully mediates the link between attachment anxiety and relationship satisfaction, and also concluded that higher PPR is an important intervention target for improving intimacy satisfaction, higher intimacy satisfaction puts the individual in secure attachment, the individual will enjoy intimate interactions with the partner and have a strong belief that the partner is reliable, and the individual will respond positively to the partner’s concerns or needs. Research has shown (Ruan et al., 2020) that an individual’s PPR is a central predictor of marital well-being. This suggests that healthcare professionals should encourage patients and their partners to emphasize the maintenance of intimacy and increase effective communication, and the partners should respond positively to needs of patients, which can enhance patients’ emotional experience and facilitate the recovery from the disease.

Intimacy was positively associated with support utilization in EM women of childbearing age in this study. A systematic review found that improving the quality of intimacy between patients with PTSD and their partners during treatment resulted in a significant increase in social support received by the patients and significantly improved treatment outcomes (Fredette et al., 2016). Kita et al. (2020) found that intimate partner violence leads to lower satisfaction with social support, feelings of loneliness, lower self-esteem, and lower support utilization in pregnant women, which can lead to depression. When individuals seek support openly, directly, and positively, it may enhance the partner’s motivation to provide support and promote individual and couple well-being (Collins and Feeney, 2000; Don et al., 2013).

Support utilization was found to mediate between intimacy satisfaction and PPR in EM women of childbearing age. Don and Hammond (2017) found that it is important to effectively seek and provide social support in an intimate relationship, the patients with higher support utilization will have better PPR. If the support provided by the giver is caring, and can help to clarify the situation and meet the needs of the recipient, it will lead to reduced stress, improved PPR and better mood in the recipient. The EM women of childbearing age are generally younger, less socially experienced, less resilient and less able to cope with stress, and they are in dire need of more positive support and affirmation from their partners. The social support utilization can exert a mediating effect between the two of them, implying that it is not only a direct factor affecting intimacy satisfaction and PPR in EM patients of childbearing age, but may also indirectly affect the overall well-being of patients by affecting the relationship between these two variables. For example, when the social support utilization is higher, the patients may be more likely to be satisfied with intimacy and perceive partner responsiveness more positively, thus creating a virtuous circle. This finding highlights the importance of social support in patients coping with the stress of illness and improving quality of life. Therefore, increasing social support utilization in EM patients may be one of the effective ways to improve intimacy satisfaction and PPR, which may also provide useful insights for medical professionals and social workers to pay attention to patients’ social support needs and provide them with necessary support and assistance while providing medical services.

## 6 Limitations and strengths

This study focuses on examining the mediating effect of social support on the relationship between intimacy and PPR in EM women of childbearing age, an aspect that has received less attention in previous studies, particularly with regard to support utilization.

Several limitations should be considered. First, recall bias should be noted in self-reported scales. Second, given the cross-sectional research design of this study, no causal inference could be established, and future longitudinal studies with stronger evidence are needed to validate such a causal relationship. Third, the subjects were collected from only one tertiary general hospital, who may not be representative of all EM women of childbearing age; therefore, the generalizability of the results of this study needs to be further verified. Fourth, this study did not assess potential confounders such as personality traits, family resilience, which may be important variables to be considered in future studies to improve model fitting.

## 7 Conclusion

Support utilization may be an important mediator between intimacy and PPR in EM women of childbearing age. The classification of social support utilization can be refined, and the specific effects of different types of social support (e.g., family support, friend support, and professional medical support) on intimacy and PPR of EM patients can be further analyzed in the future studies. The study on the mediating effect of social support utilization can provide more effective psychological intervention and social support services for EM patients, improve their quality of life, optimize the allocation of healthcare resources, enhance the harmonious development of family and society, and simultaneously promote the formulation and improvement of related policies.

## Data Availability

The original contributions presented in the study are included in the article/supplementary material, further inquiries can be directed to the corresponding author.
